# A Refractive Index Sensor Based on Spectral Interrogation of a Long Tapered Side-Hole Optical Fiber

**DOI:** 10.3390/s25164916

**Published:** 2025-08-09

**Authors:** Rafał A. Kosturek, Michał Dudek, Leszek R. Jaroszewicz

**Affiliations:** Institute of Applied Physics, Military University of Technology, 2 gen. Sylwestra Kaliskiego St., 00-908 Warsaw, Poland; michal.dudek@wat.edu.pl (M.D.); leszek.jaroszewicz@wat.edu.pl (L.R.J.)

**Keywords:** fiber optic sensor, side-hole optical fiber, tapered optical fiber, immersion liquid, refractive index

## Abstract

This article describes an external refractive index (RI) sensor based on a spectral analysis of the light transmission through a long tapered side-hole optical fiber (S-H OF). A section of the S-H OF was fusion-spliced with SMFs at both ends and connected to a supercontinuum source at the input and an optical spectrum analyzer (OSA) at the output. To investigate the effect of the external RI on the spectral characteristics, immersion liquids with refractive indices in the ranges of 1.32–1.35 and 1.37–1.42, with increments of 0.01, were used. Power loss measurements were carried out for each liquid in two wavelength ranges: 600–1200 nm and 1200–1800 nm. The highest sensitivity obtained in this study was 622 ± 11 nm/RIU in the near-infrared region. This result highlights the suitability of longtapered S-H OFs for spectral-interrogation-based RI sensing, offering a promising yet simpler alternative to more complex interferometric or plasmonic configurations.

## 1. Introduction

The growing demand for reliable, efficient, and precise environmental monitoring has driven significant advances in sensor technologies. Among these, optical fiber sensors have emerged as a key solution due to their inherent advantages, including their immunity to electromagnetic interference, compact size, and ability to operate in harsh environments [1,2,3]. Intensity-based fiber optic sensors can detect changes in light intensity resulting from variations in the surrounding environment [4,5,6]. These sensors have attracted significant attention due to their simplicity in signal processing and high sensitivity, making them particularly valuable for monitoring changes in the refractive index (RI) [7], gas concentrations [8], and biochemical factors [9,10,11]. Among the key advancements in this field, tapered optical fibers (TOFs) have emerged as a crucial element in enhancing the performance of fiber optic sensors [12,13,14]. The tapering process reduces the fiber’s diameter, modifying its optical properties to enhance the interaction between light and matter [15,16,17]. This optical modification enhances sensor sensitivity, especially for detecting subtle environmental changes. The light–matter interaction intensifies as guided light extends beyond the core into the cladding and the surrounding medium. This makes TOFs ideal for high-sensitivity applications, enabling precise detection of RI or absorbance variations. Additionally, their support for evanescent wave propagation allows for direct environmental probing, crucial for chemical and biological sensing [18,19,20,21,22,23,24,25]. Integrating side-hole optical fibers (S-H OFs) with tapering technology represents a novel approach to enhancing sensor performance [26,27,28,29,30,31]. S-H OFs inherently feature air channels adjacent to the fiber core, which offer a unique geometry for functionalization and interaction with external materials. When such fibers are tapered, the combination of a reduced diameter and the side-hole structure facilitates the generation of an intense evanescent field. This field significantly boosts the fiber’s sensitivity, allowing light to interact more effectively with the external medium. Additionally, the geometry of S-H OFs ensures mechanical stability and offers avenues for the selective introduction of materials into the holes, expanding the sensor’s applicability further [32]. By leveraging the benefits of tapered fibers and S-H OFs, researchers can achieve high sensitivity and specificity in detecting environmental changes. Recent studies have demonstrated the practical advantages of integrating tapered S-H OFs into sensor designs. In 2022, a research group led by Jinghua Fu reported a highly sensitive humidity sensor based on a fiber Mach–Zehnder Interferometer (MZI) that used a tapered dual-side-hole fiber (DSHF) [33]. The sensor was designed with a short section of tapered DSHF sandwiched between two single-mode fibers (SMFs), where both the fundamental and higher-order cladding modes were excited, forming an inter-modal MZI. The presence of air holes in the DSHF facilitated stronger excitation of the cladding modes and improved RI sensitivity. Furthermore, by coating the tapered DSHF with a graphene oxide (GO) film, which provided a large specific surface area, the researchers significantly enhanced the sensor’s sensitivity to humidity, achieving a remarkable improvement of 7.9 times compared to that in the uncoated fiber. The sensor demonstrated a sensitivity of −0.142 nm/% RH, with response and recovery times of 0.23 s and 2.19 s, respectively. Beyond humidity sensing, tapered DSHFs have also proven highly effective for measuring the RI. In 2018, a group led by Song Li reported the development of an in-line fiber MZI based on a tapered DSHF for RI measurements [26]. The MZI was constructed using a short segment of tapered DSHF, sandwiched between two SMFs. The fiber was tapered using an oxy-hydrogen flame, which allowed for precise control over its dimensions. At the taper region, the core mode of the incoming SMF was coupled to multiple cladding modes of the DSHF, leading to interference between the core and cladding modes. Due to the air holes in the tapered region, the cladding modes were more easily stimulated compared to conventional SMF-based structures. The experimental results demonstrated that the sensor exhibited an impressive RI sensitivity of 816.1 nm/RIU in the RI range of 1.333–1.379, with a fiber diameter of 33 µm. Additionally, the sensor demonstrated low thermal sensitivity, with a temperature response of only 19.9 pm/°C in the range of 30–110 °C, making it highly stable for RI measurements in varying environmental conditions. The research presented in this article focuses on the characterization of an external RI sensor based on spectral interrogation of a long-tapered S-H OF. While previous studies have employed tapered S-H OFs primarily in Mach–Zehnder configurations relying on phase differences [26,33], this work focuses on analyzing the spectral transmission characteristics, specifically the wavelength shifts in interference dips induced by changes in the external RI. While tapered optical fibers have been extensively explored for RI sensing, studies involving tapered side-hole optical fibers remain limited—particularly with regard to their direct spectral response. So far, no studies have been reported on long tapered S-H OF structures with extended taper waists. This study aims to address this gap by experimentally characterizing their interference-based sensitivity across a broad RI range and two distinct spectral regions.

## 2. Materials and Methods

**Figure 1:**
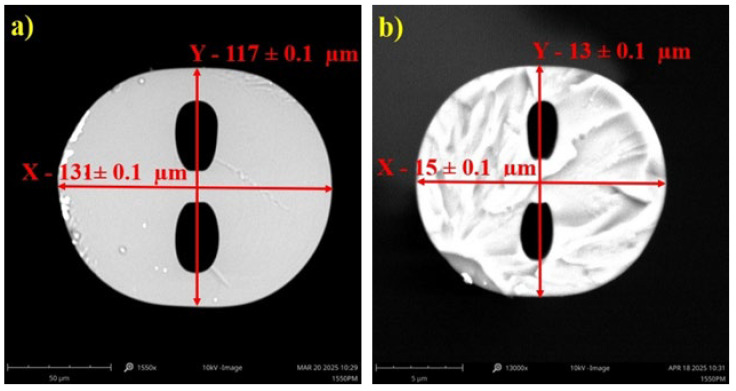
(**a**) The adopted coordinate system in the cross-section of the S-H OF. (**b**) The tapered S-H OF structure.

The TOFs used in this research were fabricated from S-H OF with an elliptical core measuring 2 × 4 ± 0.1 μm and air holes with dimensions of 20 × 35 ± 0.1 μm. The length of the y-axis cross-section was 117 µm, while for the x-axis cross-section, it was 131 µm (Figure 1a). The fiber with this geometry was manufactured by the Fiber Optics Technology Laboratory at Maria Curie-Skłodowska University in Lublin, Poland. The tapering process was performed using a Fiber Optic Tapered Element Technology (FOTET), a system extensively described in previous studies [34,35]. The movable burner, an integral part of the FOTET system, enables the fabrication of tapers with varying taper waist lengths. During this research, 15, 20, and 25 mm tapers were successfully fabricated on the S-H OF while preserving their internal structure. The geometry of the taper was carefully selected based on preliminary tests indicating that a 20 mm taper offered the most balanced compromise between sensitivity and mechanical stability. The 15 mm tapers exhibited relatively low sensitivity to changes in the external RI, likely due to insufficient modal interference, while the 25 mm structures, although more responsive, showed reduced mechanical stability as a result of excessive waist thinning. The manufactured tapered S-H OF was characterized by an elongation of 20.00 ± 0.05 mm. After the tapering process, the dimensions of the S-H OF were approximately 13 µm along the y-axis and 15 µm along the x-axis, confirming the preservation of the fiber’s ellipticity. The internal holes were characterized by estimated dimensions of 3.8 µm in the y-axis and 1.95 µm in the x-axis, indicating a tenfold reduction in the internal geometric elements. The core dimensions were estimated at 0.2 µm along the x-axis and 0.4 µm along the y-axis. The tapered fiber with this geometry is shown in Figure 1b.

**Figure 2:**
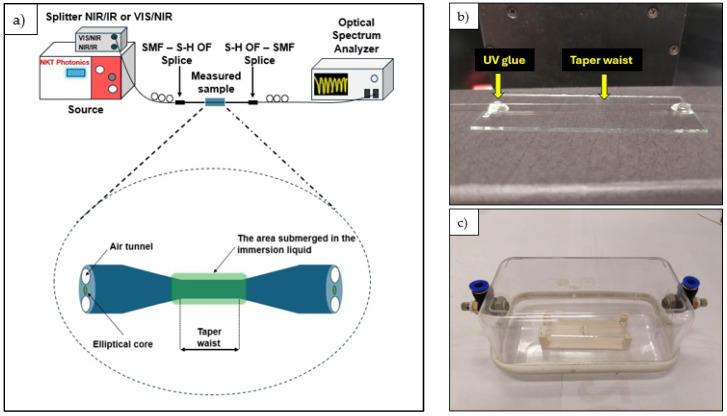
(**a**) The measurement setup. (**b**) The tapered S-H OF mounted onto a glass plate using UV glue. (**c**) A photo of a sample placed inside a temperature shielding chamber.

The section of S-H OF containing the tapered region was mounted onto a glass plate using UV-curable adhesive applied to both ends of the fiber segment. The tapered region had no contact with the UV adhesive. In the next step, the tapered S-H OF was fused to standard SMFs at both ends using a VYTRAN FFS-2000WS splicer (Thorlabs, Morganville, NJ, USA). Due to the mismatch in the core dimensions between the S-H OF and SMF, the possibility of using polymer connectors was taken under consideration [36,37,38], but fusion splicing proved to be more effective and easier to perform in this case. The assembled SMF–S-H OF structure was then connected via FC/PC connectors, with one end linked to an SuperK Compact ( NKT Photonics, Birkerod, Denmark) supercontinuum source and the other to a Yokogawa AQ6370D (Yokogawa Meters & Instruments Corporation, Tokyo, Japan)optical spectrum analyzer (OSA). The OSA operated in the 600–1200 nm and 1200–1800 nm ranges, configured with a resolution of 1 nm. The complete experimental setup, including a close-up of the tapered region mounted to a glass plate and a photograph of a sample placed in a thermally shielded chamber, is presented in Figure 2. Subsequently, an immersion liquid with RI values from 1.32 to 1.42 (in increments of 0.01) was applied to the surface of the tapered structure.

The immersion liquid was applied using a syringe to ensure full coverage of the taper waist area. After its application, its impact on the spectral characteristics was analyzed. The liquid was then removed using 99.8% isopropanol until the optical power returned to its reference level before application. All measurements were conducted at a controlled ambient temperature of 22 ± 1 °C. Additional tests were conducted to evaluate the thermal stability of the interference pattern. Additional thermal tests were carried out to verify the stability of the sensor’s spectral response under elevated temperatures. The clean, uncoated tapered fiber was exposed to temperatures of up to 50 °C, and no meaningful changes in the transmission spectrum were observed. These results confirmed the structural and spectral stability of the sensor within this thermal range. Nonetheless, all of the experimental measurements presented in this work were conducted at a controlled room temperature of 22 ± 1 °C to minimize any thermal influence. The RIs of the immersion liquids (Cargille Labs) were taken from the manufacturer’s specifications, calibrated at the sodium D line (589.3 nm) for a standard temperature of 25 °C, and recalculated for two wavelengths—632 nm and 1310 nm—depending on the investigated range. The RI = 1.36 data point was excluded from the final analysis due to measurement instability, which was likely caused by contamination or degradation of the immersion liquid, leading to inconsistent spectral responses. Although the S-H OF used in this work was polarization-maintaining by design, polarization effects were not considered in this study. Since a supercontinuum light source with an unpolarized output was used, no polarization control was applied at the input, and the measurements represent the averaged response over all polarization states. To ensure the reliability of the presented data, multiple tapered S-H OF samples were fabricated and tested. Although all samples exhibited modal interference patterns and RI-dependent shifts, the results discussed in this paper correspond to the most representative structure. This particular sample demonstrated the most stable spectral response and the lowest splice losses. Each measurement for this sample was repeated three times under identical conditions, yielding consistent and reproducible spectral results.

## 3. Results

**Figure 3:**
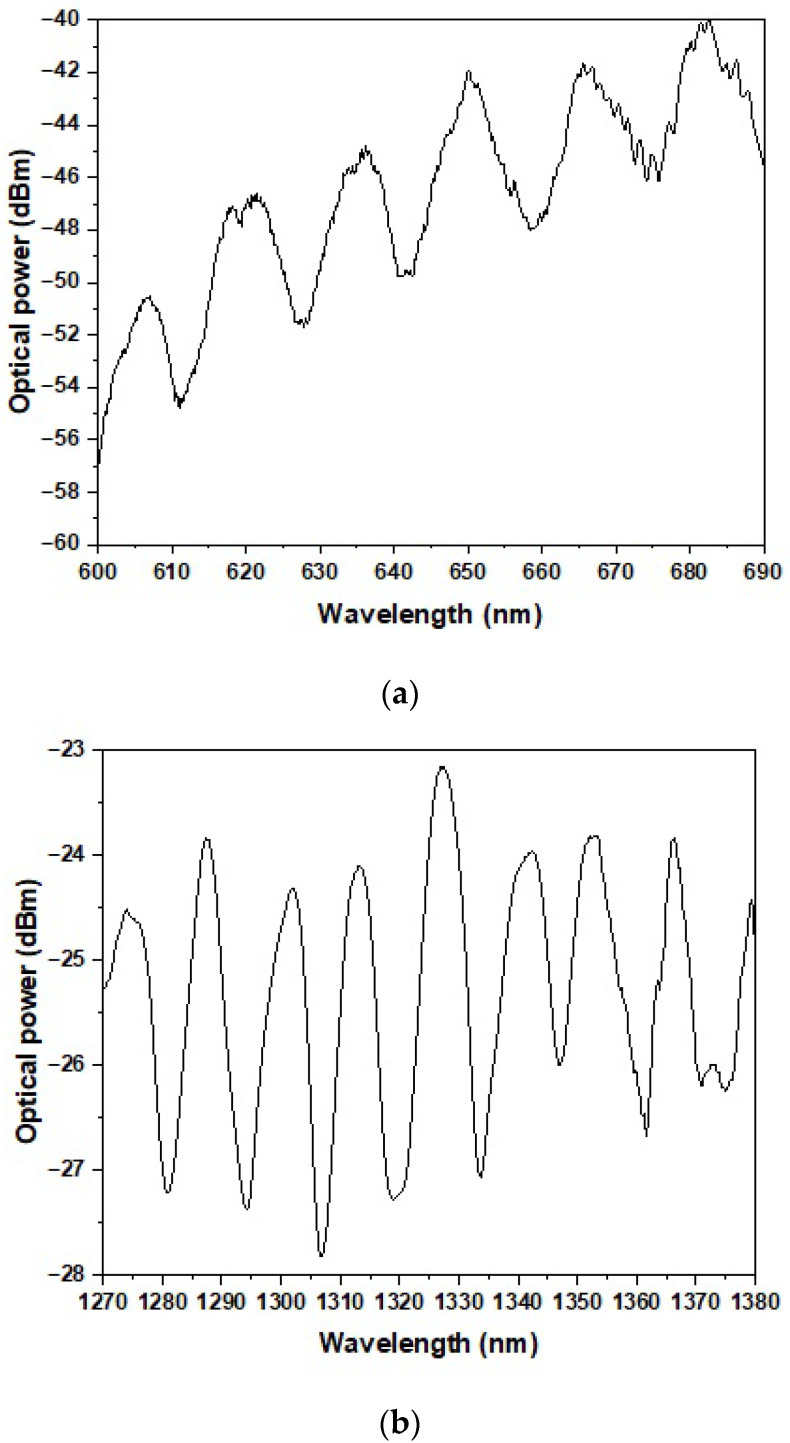
The reference optical power for the SMF–S-H OF–SMF connection in the wavelength ranges of (**a**) 600–690 nm and (**b**) 1270–1380 nm.

In the first step, the reference power was measured for the spliced SMF-S-H OF connection in two broad wavelength ranges of 600–1200 nm and 1200–1800 nm. The observed interference pattern in the transmission spectrum, particularly visible in the ranges of 600–690 nm and 1270–1380 nm (Figure 3), is attributed to modal interference occurring within the tapered fiber structure, most likely between the fundamental core mode and the cladding modes excited in the taper waist. The spectral positions of the interference dips are sensitive to the external RI due to the interaction of the cladding modes’ evanescent field with the surrounding medium. The dips recorded for the reference optical power level were located within the optical loss range of −40 to −60 dBm for the wavelength range of 600–690 nm and −23 to −28 dBm for the range of 1270–1380 nm. The high optical losses observed in the 600–690 nm range were caused by the use of two fiber loops placed along the optical path in the input SMF to radiate higher-order modes and ensure single-mode propagation in this wavelength range.

**Figure 4:**
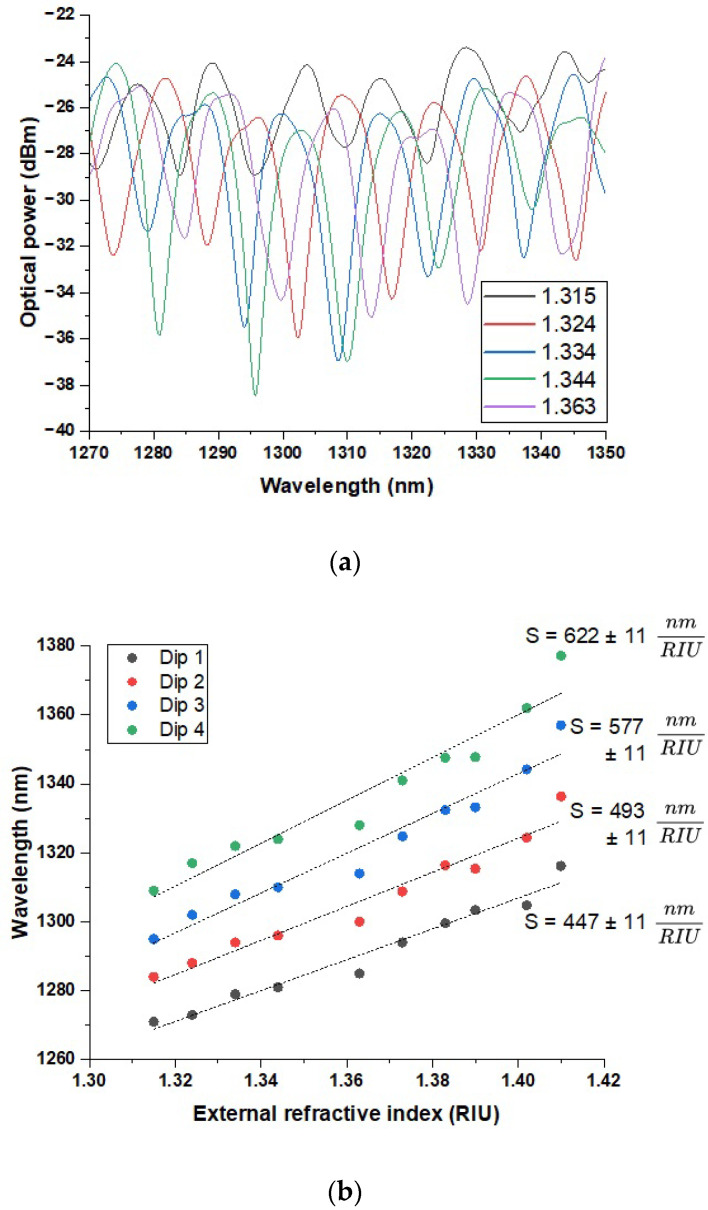
(**a**) The obtained spectral response for immersion liquids with five representative RI values in the wavelength range of 1270–1350 (the wavelength range of 1270–1350 nm is displayed for improved clarity of the spectral features). (**b**) A graph illustrating the dip positions in the wavelength range of 1270–1380 nm depending on the given RI values in the 1.315–1.41 range.

In the next step, the influence of applying immersion liquids was examined using an OSA operating in the 1200–1800 nm range. A graph of the optical power versus the wavelength was obtained for immersion liquids with the following RI values (recalculated for λ = 1310 nm): 1.315, 1.324, 1.334, 1.344, 1.363, 1.373, 1.383, 1.39, 1.402, and 1.41. The results revealed the presence of characteristic dips in the λ = 1270–1380 nm range, which appeared to shift toward longer wavelengths as the RI value increased. Figure 4a presents the results for five representative RI values. The first dip exhibits a wavelength shift beginning at λ = 1271 nm for an RI = 1.315 and extending to λ = 1316 nm for an RI = 1.41. Based on these values, the sensor’s sensitivity *S* was estimated to be 447 ± 11 nm/RIU. For the second dip, the shift ranges from λ = 1284 nm to λ = 1336 nm across the same RI interval, corresponding to a sensitivity of 493 ± 11 nm/RIU. According to the wavelength shift measurements for the third dip, ranging from λ = 1295 nm at an RI = 1.315 to λ = 1357 nm at an RI = 1.41, the sensor exhibited a sensitivity of 577 ± 11 nm/RIU. Similarly, the data obtained for the fourth and final dip revealed a spectral shift from λ = 1309 nm to λ = 1377 nm across the same RI range, corresponding to a sensitivity of 622 ± 11 nm/RIU. All sensitivity values were derived from the slopes of linear approximations fitted to the recorded data (Figure 4b).

**Figure 5:**
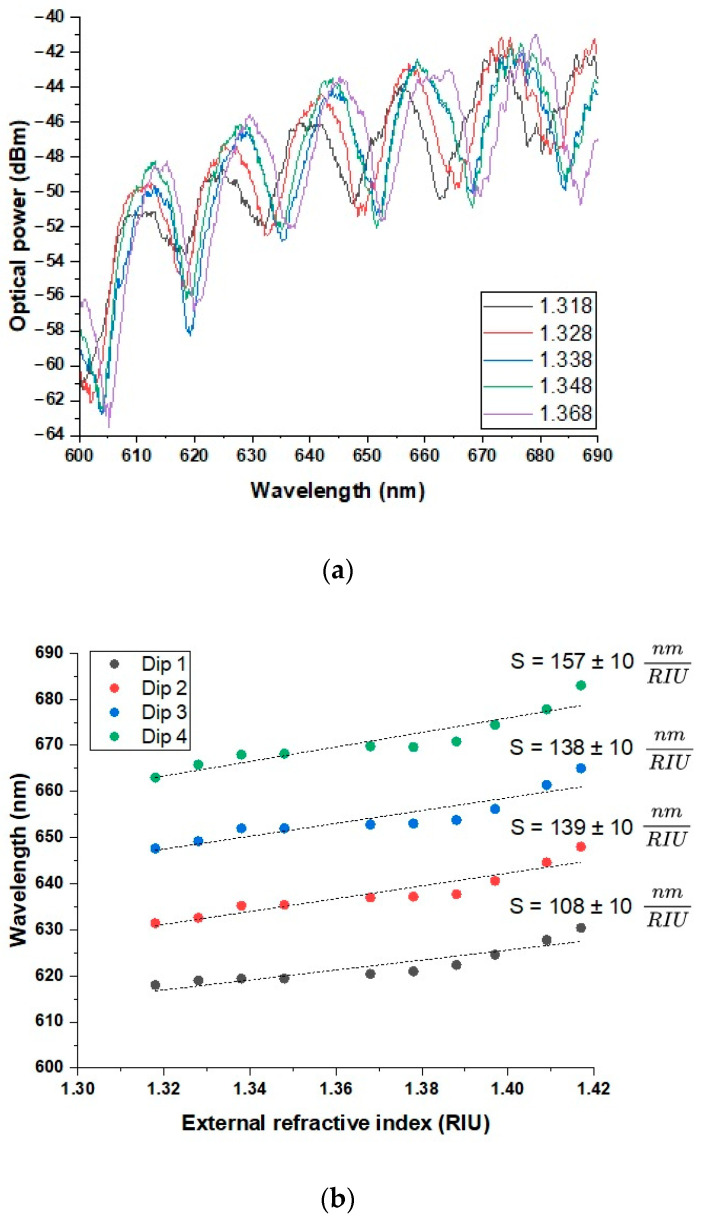
(**a**) The obtained spectral response for immersion liquids with five representative RI values in the wavelength range of 600–690 nm. (**b**) A graph illustrating the dip positions in the wavelength range of 600–690 nm depending on the given RI values in the 1.318–1.417 range.

In the next phase of this study, an identical analysis was carried out within the 600–1200 nm spectral range for immersion liquids with the following RI values (recalculated for λ = 632 nm): 1.318, 1.328, 1.338, 1.348, 1.368, 1.378, 1.388, 1.397, 1.409, and 1.417. As in the earlier case, the findings revealed a shift in the interference dips toward longer wavelengths with an increasing RI of the applied immersion liquid. Figure 5a) illustrates the results for five selected RI values within the most distinct wavelength region, spanning 600–690 nm. Within this range, the initial dip was observed to move from 618 nm at an RI = 1.318 to 630.4 nm at an RI = 1.417. An approximation of these values yielded a sensitivity of 108 ± 10 nm/RIU. For the second dip, located at 632 nm for the lowest RI and shifting to 648 nm at the highest, the calculated sensitivity reached 139 ± 10 nm/RIU. Similarly, the third dip transitioned from 648 nm to 665 nm as the RI increased, resulting in a sensitivity of 138 ± 10 nm/RIU, practically identical to the value determined for the previous one. Finally, the fourth and last observed dip shifted from 663 nm to 683 nm over the same RI interval, corresponding to a sensitivity of 157 ± 10 nm/RIU. As with the previous spectral range, each sensitivity value was determined based on the slope of the linear trend lines fitted to the measurement data (Figure 5b).

## 4. Discussion

The experimental results obtained for the tapered side-hole optical fiber sensor revealed distinct interference patterns in both spectral ranges analyzed, with the interference dips shifting progressively toward longer wavelengths as the external RI increased. This behavior confirms the sensor’s capability to operate based on spectral interrogation principles and highlights its sensitivity to changes in the surrounding medium. Figure 3 presents the reference transmission spectra for the fabricated SMF–S-H OF–SMF configuration, demonstrating the presence of interference dips attributed to modal interference between the fundamental mode and the cladding modes in the taper region. These features served as baselines for the RI-induced spectral shifts. The main sensing experiments conducted in the 1270–1380 nm range (Figure 4) and the 600–690 nm range (Figure 5) showed that for both regions, increasing RI values consistently resulted in a shift in the observed interference minima toward longer wavelengths. In the near-infrared range, four distinct dips were tracked, yielding sensitivities of 447 ± 11, 493 ± 11, 557 ± 11, and 622 ± 11 nm/RIU. In the visible and near-infrared range, the sensitivities associated with the four corresponding dips were calculated as 108 ± 10, 139 ± 10, 138 ± 10, and 157 ± 10 nm/RIU. These results, presented in Figure 4b and Figure 5b, clearly demonstrate a near-linear relationship between the dip position and the RI, confirming the sensor’s reliability and facilitating potential calibration for practical applications. The significantly higher sensitivity values in the 1270–1380 nm region reflect the physical principle that longer wavelengths support a more extensive evanescent field, thereby enhancing light–matter interactions in the surrounding medium. This trend confirms previously reported observations in tapered fiber sensors and suggests that the use of longer operating wavelengths could be strategically employed to increase sensor performance. It is worth noting that each interference dip exhibits distinct sensitivity to changes in the external RI. This behavior can be attributed to the modal nature of the observed interference: each dip arises from the interaction between the fundamental core mode and a particular cladding mode. As demonstrated in Turan Erdogan’s analysis of cladding mode resonances, each cladding mode has a different effective RI and modal field distribution [39]. These differences affect the degree of evanescent field penetration into the surrounding medium and consequently the mode responsiveness to RI changes. Modes with greater extension of the field into the external environment contribute to higher sensitivity. An important design aspect confirmed by this study is the viability of using a long taper (20 mm) on an S-H OF to achieve sufficient modal interference for RI detection without relying on explicit interferometric structures such as an MZI. The presence of interference dips and their systematic shifts validates the effectiveness of this simple yet sensitive configuration. The decision to exclude the RI = 1.36 data point due to unstable results further underscores the importance of measurement repeatability and fluid purity. Such instability could stem from liquid contamination, degradation, or surface adhesion inconsistencies, all of which are known to affect evanescent-field-based sensors. Overall, these results indicate that the proposed sensor configuration, while simpler than many interferometric or plasmonic systems, provides competitive sensitivity values and offers potential for further enhancements through surface functionalization or integration with selective coatings. Additionally, to evaluate the resolution capability of the sensor, the *Q* factor and the limit of detection (*LOD*) were calculated for the most prominent interference dip. The *Q* factor is defined as the ratio of the resonance wavelength ($\lambda_{res}$) to the full width at half maximum (*FWHM*):

(1) $$Q=\frac{\lambda_{res}}{FWHM}$$

The *LOD* is then given by

(2) $$LOD=\frac{\lambda_{res}}{Q\cdot S}$$

For the resonance dip located at $\lambda_{res}$= 1316 nm, with an *FWHM* of 4 nm and a sensitivity *S* = 622 nm/RIU, the calculated *Q* factor is 329. The resulting *LOD* is approximately 6.4 × 10^−3^ RIU. This value indicates the minimum RI change detectable by the sensor and further confirms its applicability in high-resolution RI monitoring. In the visible range, the *LOD* was also estimated for the most sensitive dip—the fourth dip described in the manuscript—based on its experimentally observed shift and sensitivity. For this dip, a resonance position of $\lambda_{res}$ = 665 nm, an FWHM = 6 nm, and a sensitivity of 157 nm/RIU were obtained. Under these conditions, the calculated *Q* factor is 111, and the corresponding *LOD* is approximately 38.3 × 10^−3^ RIU. When compared to the *Q* factor (329) and the *LOD* (6.43 × 10^−3^ RIU) of the most sensitive dip in the near-infrared region, it becomes evident that the sensor performs significantly better in the infrared domain. This improvement is primarily due to the narrower interference dips observed in the near-infrared spectrum, which contribute directly to higher *Q* values and improved resolution. Therefore, for applications requiring precise detection of small RI changes, this sensor configuration is better suited to operation in the infrared range.

## 5. Conclusions

This work demonstrated the feasibility of using a long tapered S-H OF for external RI sensing based on spectral interrogation. The fabricated sensor exhibited clear interference patterns and a linear spectral response to changes in the surrounding RI across two distinct spectral ranges. The highest sensitivity, recorded in the 1270–1380 nm range, reached 622 ± 11 nm/RIU, a value that confirms the suitability of this structure for high-resolution RI detection. Although the sensitivity achieved is lower than that of certain specialized interferometric or plasmonic fiber sensors, the simplicity of the design, absence of additional coatings or interferometric arms, and relatively low fabrication complexity make this approach attractive for a variety of sensing applications. A comparison with representative RI sensors reported in the literature is presented in Table 1, highlighting the relative performance and practical advantages of the proposed configuration.

Given its relatively high sensitivity in the near-infrared range and straightforward fabrication process, the developed sensor based on a long tapered S-H OF shows strong potential for practical implementation. Its ability to monitor RI changes in real time makes it particularly suitable for applications in chemical processing, environmental analysis, and liquid quality control. Notably, as shown in Table 1, the sensor achieves a superior sensitivity compared to that of many previously reported taper-based configurations (e.g., SMF or photonic crystal fibers) and offers an extended RI measurement range, which exceeds that of several high-sensitivity designs. The tested RI range encompasses a wide spectrum of real-world liquid media, including aqueous ethanol solutions, methanol–water mixtures, and various organic solvents such as acetone (RI ≈ 1.359), isopropanol (RI ≈ 1.377), and ethyl acetate (RI ≈ 1.372), all of which are commonly encountered in environmental samples, industrial processing, and biochemical applications. In addition, the platform’s compatibility with surface functionalization techniques allows for future adaptation to selective detection tasks, including gas sensing for analytes such as ammonia or ethanol in complex environments.

## Figures and Tables

**Table 1:** External RI sensitivity for different TOF structures.

Structure	RI Range	Sensitivity [nm/RIU]	Ref.
Tapered DSHF	1.333–1.379	~861.1	[26]
Tapered SMF	1.333–1.403	~489.8	[40]
Double-tapered photonic crystal fiber	1.333–1.373	~281.6	[41]
Optical fiber with removed cladding	1.36–1.43	~471	[42]
Long-tapered S-H OF	1.315–1.41	~622	This work

## Data Availability

The data is available upon request due to the volume of data, as well as project limitations.
